# Supplementary material for: Characterization of a Unique Pair of Ferredoxin and Ferredoxin NADP+ Reductase Isoforms That Operates in Non-Photosynthetic Glandular Trichomes
Source: Plants (Basel). 2024 Jan 30;13(3):409. doi: 10.3390/plants13030409 (PMC10857128; doi:10.3390/plants13030409)
Supplement: Supplementary file 1 [file plants-13-00409-s001.zip › plants-2794334-supplementary.pdf]

Supplementary Materials

Table S1. List of primers used to amplify and clone *Fd* and *FNR* genes from first-strand cDNA into a pSBET expression vector. Plastidial targeting sequences are excluded to generate amplicons that correspond to the native proteins.

Gene	Primer ID	Primer Forward (5'-3')	Primer Reverse (5'-3')
FNR (Leaf)	L-FNR-A	TGGCACAGGTAACCACAGAAGCAC	CTCAGTACACTTCCACATTCCACTG
FNR (Leaf)	L-FNR-B	TATGGCACAGGTAACCACAGAAGCAC	GATCCTCAGTACACTTCCACATTCCACTG
FNR (GT/Root)	R-FNR-A	TGTCAGTGCAACAAGCTAGC	CTTAGTAGACTTCAACATGCCA
FNR (GT/Root)	R-FNR-B	TATGTCAGTGCAACAAGCTAGC	GATCCTTAGTAGACTTCAACATGCCA
Fd I (Leaf)	Fd-1-A	TGGCTAGCTACAAGGTGAAGCTG	CTCAAGCAATATCATCCTCCTTGTGCTG
Fd I (Leaf)	Fd-1-B	TATGGCTAGCTACAAGGTGAAGCTG	GATCCTCAAGCAATATCATCCTCCTTGTGCTG
Fd II (Major GT/Root)	Fd-2-A	TGGCTGTATACAAGGTGAAAC	CTCAGTAGAGATCACCCTCC
Fd II (Major GT/Root)	Fd-2-B	TATGGCTGTATACAAGGTGAAAC	GATCCTCAGTAGAGATCACCCTCC
Fd III (Minor GT/Root)	Fd-3-A	TGGCCACATACAAGGTGAAATTG	CTCAATACAAATCACTTTCCTTGTG
Fd III (Minor GT/Root)	Fd-3-B	TATGGCCACATACAAGGTGAAATTG	GATCCTCAATACAAATCACTTTCCTTGTG

Table S2. List of peppermint genes and their respective primers that were used in the qPCR analysis.

Gene	Type	Name	qPCR FWD (5'-3')	qPCR REV (5'-3')
GAPDH	Reference	Glyceraldehyde-3-Phosphate Dehydrogenase	AGGCATCCTTCGGTCTAAATG	TGTGTCGCCGTTGCTTTA
ACT	Reference	Actin	TGTCAGCAACTGGGATGATATG	CGATTGGCCTTGGGATTAAGA
CYP	Reference	Cyclophilin	CGGAAGGATCGTGATGGAG	GAAGCACGATCCCTTGTAGT
EIF3E	Reference	Eukaryotic Translation Initiation Factor 3E	TCGCACTTGAGGAGCTTAAC	CGTTGTCATGGTTGAAGAAGATG
PP2A	Reference	Serine/threonine-protein phosphatase 2A	CTGGAGGTCATGTACCAGAAAC	CCACGTAGGAGAGTAATGTTAGC
α-TUB	Reference	Alpha Tubulin	TGACACGTCTGTCGGAATTG	CCAGTCCTAACTTCGTCGATAAC
Fd III + PT	Experimental	Ferredoxin III (Minor) (with plastidial targeting sequence)	TCACGACTGCACCTCTGA	TGGCCATTGCAGAAGCTC
Fd II + PT	Experimental	Ferredoxin II (Major) (with plastidial targeting sequence)	CAAAAGTGCTCCTCAAAGGC	GGCAGTTGCTTTGAAATCGG
Fd I	Experimental	Ferredoxin I (Leaf)	ACAAGGTGAAGCTGGTGAC	AGGTGGAGCAGGATCCG
R-FNR	Experimental	Ferredoxin NADP-Reductase (from GTs / roots)	CACCCTTAGCAGAGAACAGAAG	CTTGAGCCCACAGAAGTAGATG
L-FNR	Experimental	Ferredoxin NADP-Reductase (from leaves)	GCTCCTTTCCGCTCTTTCTT	GCTCGAATTCCTCCTTGTAGAG

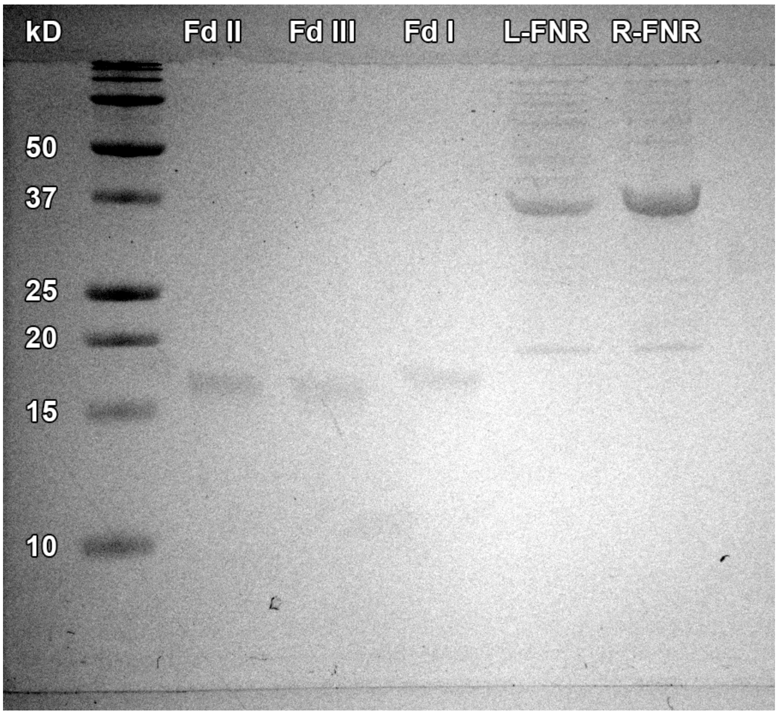

Figure S1. Tricine SDS-PAGE (16 %) of purified recombinant Fd and FNR isoforms from peppermint. All experimental lanes contain 20 µg of protein as determined by the Bradford protein assay. The ladder lane (left) contains 3.5 µl of Precision Plus Protein Standard (Cat. No. 161-0373, Bio-Rad, Hercules, CA, USA).

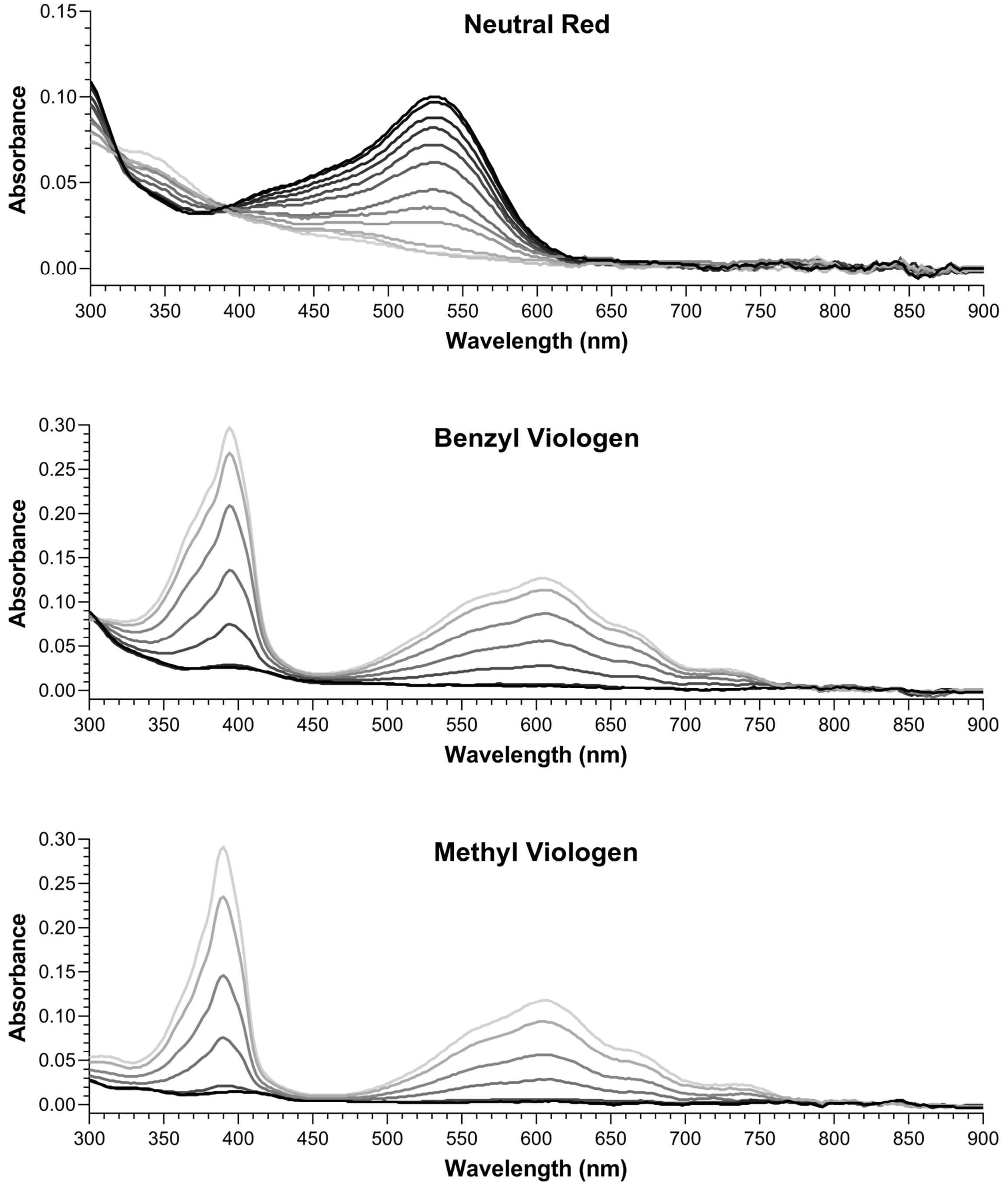

Figure S2. Absorbance spectrum of individual redox dyes subjected to photoreduction for determination of isosbestic points. Black lines are most oxidized and light-gray lines are most reduced. Top panel, neutral red; middle panel, benzyl viologen; bottom panel, methyl viologen.

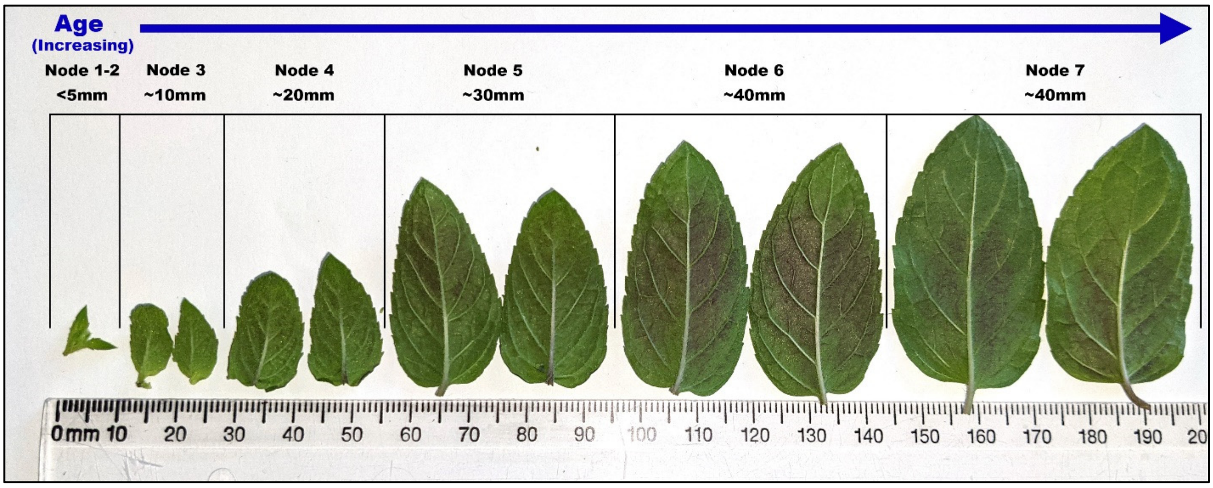

Figure S3. Peppermint leaves in order of increasing developmental age and their respective leaf blade lengths (measured from the tip of the leaf to point of petiole attachment). Leaves are grouped in pairs based on their stem attachment node and increasing distance from the meristem.

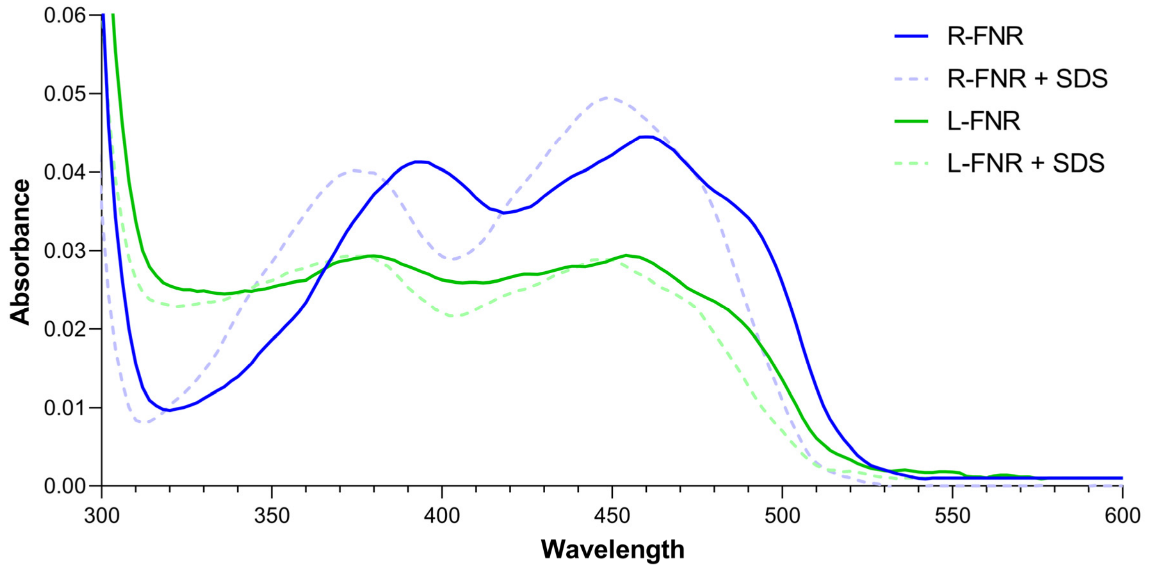

Figure S4. Characteristic absorbance spectra of purified, recombinant FNR isoforms from peppermint (diluted 1:10 from stock in 10 mM HEPES buffer at pH 7.0). Solid lines show FNR isoforms without SDS denaturation with λ_{max} at 392 nm and 460 nm for R-FNR (blue) and 380 nm and 456 nm for L-FNR (green). Dashed lines show FNR isoforms denatured by the addition of SDS up to 0.2%, demonstrating the characteristic absorbance spectra of the now dissociated FAD cofactor with λ_{max} shifted to 375 nm and 450 nm for both isoforms.

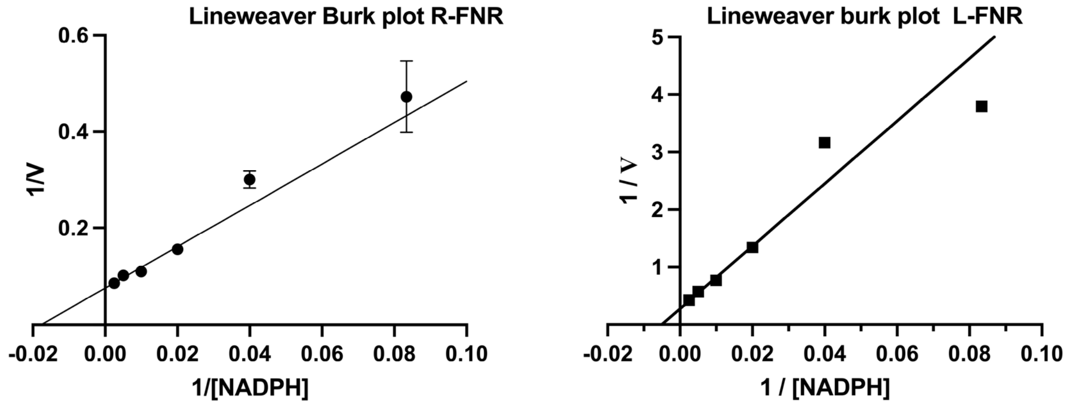

Figure S5. Potassium ferricyanide diaphorase Lineweaver-Burk plots for 0.05 µM R-FNR and 0.05 µM L-FNR from peppermint paired with variable concentrations of NADPH.

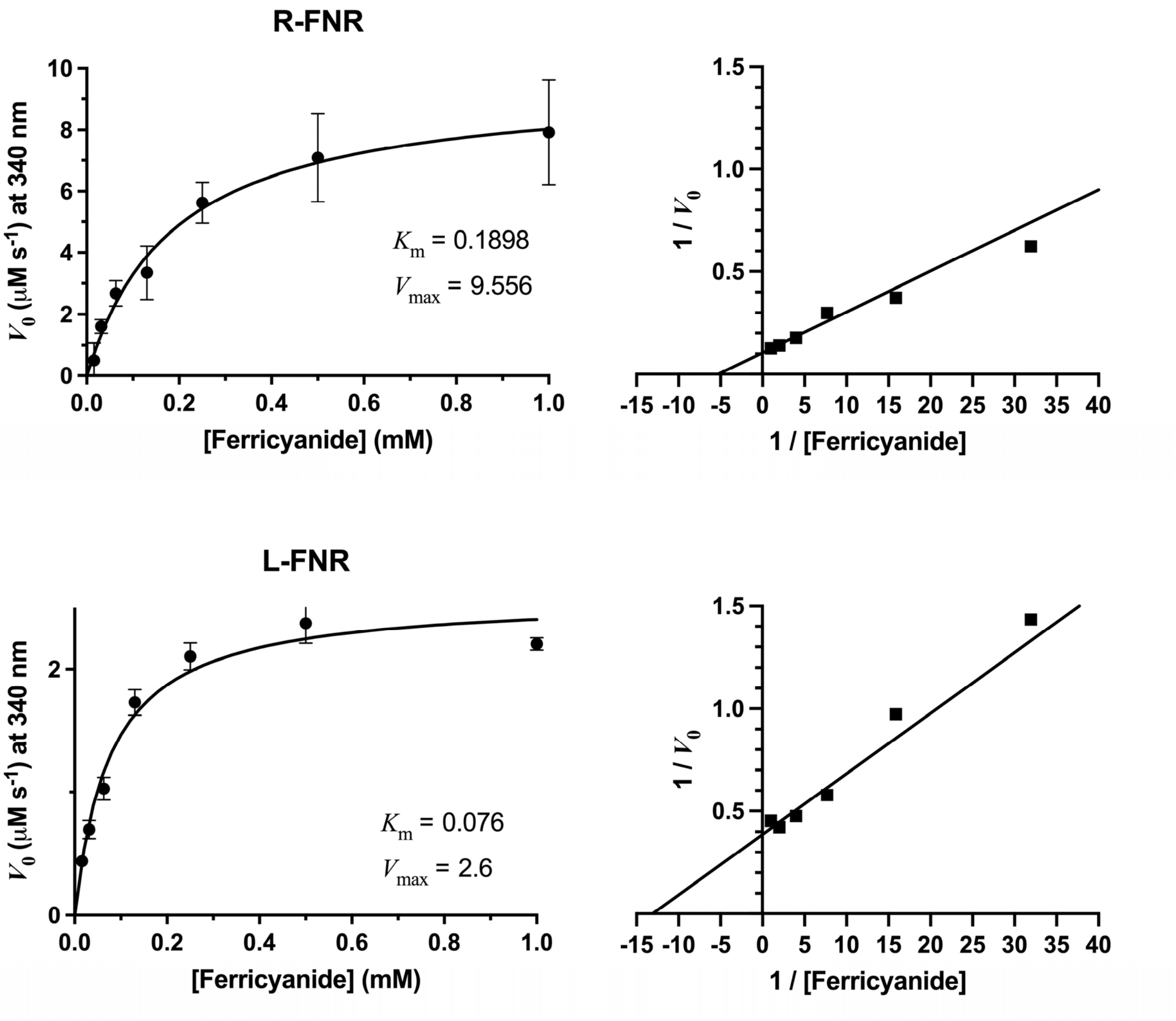

Figure S6. Potassium ferricyanide diaphorase Michaelis-Menten curves (left side) and Lineweaver-Burk plots (right side) for 0.05 μM R-FNR and 0.05 μM L-FNR from peppermint paired with variable concentrations of potassium ferricyanide.

```
                            10         20         30         40         50         60
                    ....|....| ....|....| ....|....| ....|....| ....|....| ....|....|
R-FNR               MAHSVLSQVP VAVSVNNDVS LRK-SVFKSN HVSFHEKSWS SSLSMDFRIS SFQSKGRPT-  58
L-FNR               MTAAVSAAVS LPSSKSTSLT ART-SIVSPE RIHFNK---- --FPLHYRNV AK---IVPIR  50
L-FNR-spinach       MTTAVTAAVS FPSTKTTSLS ARSSSVISPD KISYKK---- --VPLYYRNV SATGKMGPIR  54
Clustal Consensus   *: :* : *  .  : ...::  *. *:.. : :: :::       . : :*   :      *    23

                            70         80         90        100        110        120
                    ....|....| ....|....| ....|....| ....|....| ....|....| ....|....|
R-FNR               --VCMSVQQA SKPKVAVSPL SLEDAKDPPL HLFKNKEPYT GTIVSVERIV GENAPGETCH  116
L-FNR               AQVT------ TEAPAKAEKI HKKQEEGIIV NKFRPKEPYV GRCLLNVRLT GDDAPGETWH  104
L-FNR-spinach       AQIASDVEAP PPAPAKVEKH SKKMEEGITV NKFKPKTPYV GRCLLNTKIT GDDAPGETWH  114 (59)
Clustal Consensus     :            . ..     :  :.  : : *: * **. *  :   ::. *::***** *  46

                           130        140        150        160        170        180
                    ....|....| ....|....| ....|....| ....|....| ....|....| ....|....|
R-FNR               IVIDHDGKVP YWEGQSYGII PPGENPKKPG NPHNVRLYSI ASTRYGDFFD GKTASFCVRR  176
L-FNR               MVFSTEGEVP YREGQSIGII PDGID--KNG KPHKLRLYSI ASSALGDFGD SKTVSLCVKR  162
L-FNR-spinach       MVFSHEGEIP YREGQSVGVI PDGED--KNG KPHKLRLYSI ASSALGDFGD AKSVSLCVKR  172 (117)
Clustal Consensus   :*:. :*::* * **** *:* * * :  * * :**::***** **:  *** * .*:.*:**:*  92

                           190        200        210        220        230        240
                    ....|....| ....|....| ....|....| ....|....| ....|....| ....|....|
R-FNR               AVYYDPETGK EDPSKKGVCS NFLCDSKPGD KVQITGPSGK IMLLPENDPN ATHIMIATGT  236
L-FNR               LIYTND---- AGEVVKGVCS NFLCDLKAGA EVKITGPVGK EMLMPK-DPN ATIIMLGTGT  217
L-FNR-spinach       LIYTND---- AGETIKGVCS NFLCDLKPGA EVKLTGPVGK EMLMPK-DPN ATIIMLGTGT  227 (172)
Clustal Consensus    :* :       .   ***** ***** * *  :*::*** **  **:*: *** ** **:.***  132

                           250        260        270        280        290        300
                    ....|....| ....|....| ....|....| ....|....| ....|....| ....|....|
R-FNR               GVAPYRGYLR RMFMENVPNF KFGGLAWLFL GVANKDSLLY DEEFSNYLQE YPDNFRFDRT  296
L-FNR               GIAPFRSFLW KMFFEEHEDY KFNGLAWLFL GVPTGSSLLY KEEFELMKEK NPENFRLDFA  277
L-FNR-spinach       GIAPFRSFLW KMFFEKHDDY KFNGLAWLFL GVPTSSSLLY KEEFEKMKEK APDNFRLDFA  287 (232)
Clustal Consensus   *:**:*.:*  :**:*:  :: **.******* ** . .**** .***.   ::  *:***:* :  176

                           310        320        330        340        350        360
                    ....|....| ....|....| ....|....| ....|....| ....|....| ....|....|
R-FNR               LSREQKNKSG GKMYVQDKIE EYSDEIFKLL DN-GAHIYFC GLKGMMPGIQ DTLKKVAELR  355
L-FNR               VSREQTNAKG EKMYIQTRMA EYAEELWEML KKDNTFVYMC GLKGMEKGID DIMVSLAARD  337
L-FNR-spinach       VSREQTNEKG EKMYIQTRMA QYAVELWEML KKDNTYFYMC GLKGMEKGID DIMVSLAAAE  347 (292)
Clustal Consensus   :****.* .*  ***:* ::  :*: *::::* .: .:..*:* *****  **: * : .:*     216

                           370        380
                    ....|....| ....|....| ..
R-FNR               GESWEEKLSQ LKKNKQWHVE VY 377
L-FNR               GIDWIEYKRQ LKKAEQWNVE VY 359
L-FNR-spinach       GIDWIEYKRQ LKKAEQWNVE VY 369 (314)
Clustal Consensus   * .* *   * *** :**:** ** 231
```

Figure S7. Alignment of FNR sequences. Residue numbers in parentheses are counted after removal of the plastidial targeting sequence. Legend for consensus: "*" means that the residues in that column are identical in all sequences in the alignment; "**:**" means that conserved substitutions have been observed; "**.**" means that semi-conserved substitutions are observed. Conserved means the amino acid is replaced by one having similar characteristics.

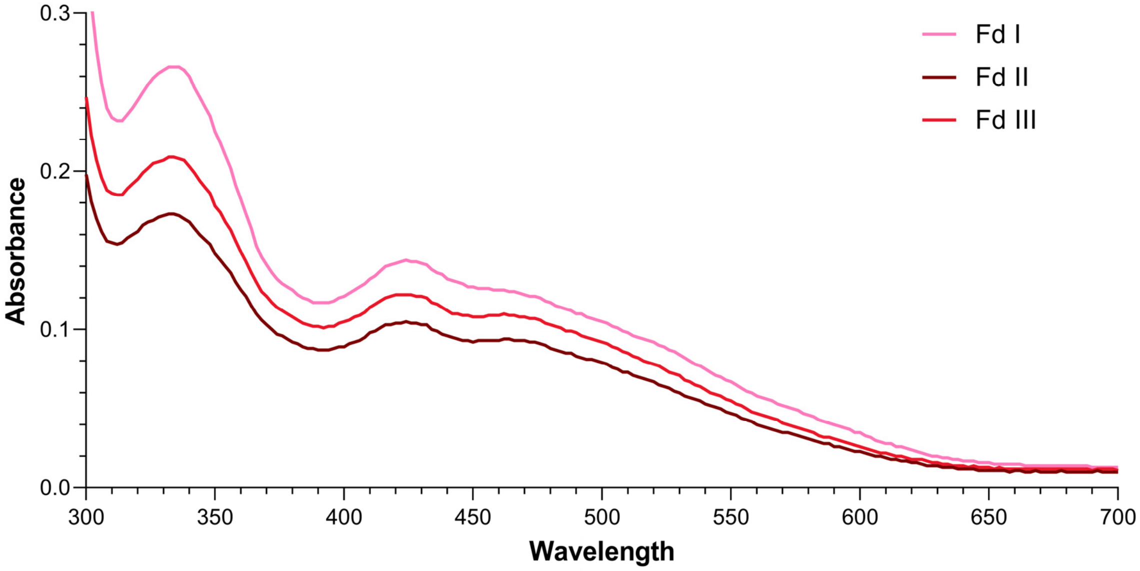

Figure S8. Characteristic absorbance spectra of purified recombinant Fd isoforms from peppermint (diluted 1:20 from stock in 50 mM Tris buffer at pH 7.5) with λ_{max} at 330 nm and 420 nm for all isoforms.

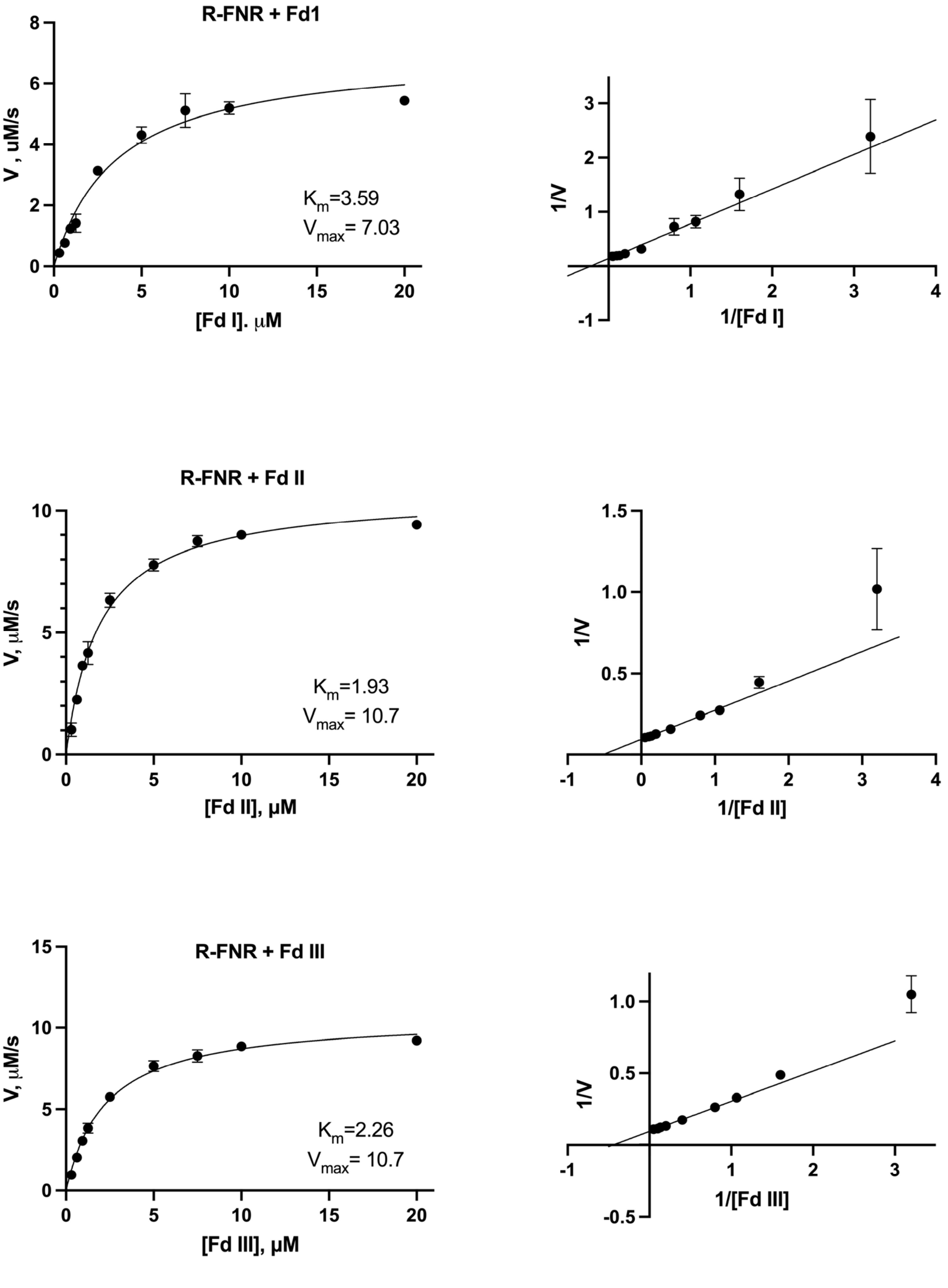

Figure S9. Cytochrome c reduction Michaelis-Menten curves (left side) and Lineweaver-Burk plots (right side) for 0.025 μM R-FNR paired with variable concentrations of Fd I, Fd II, and Fd III from peppermint.

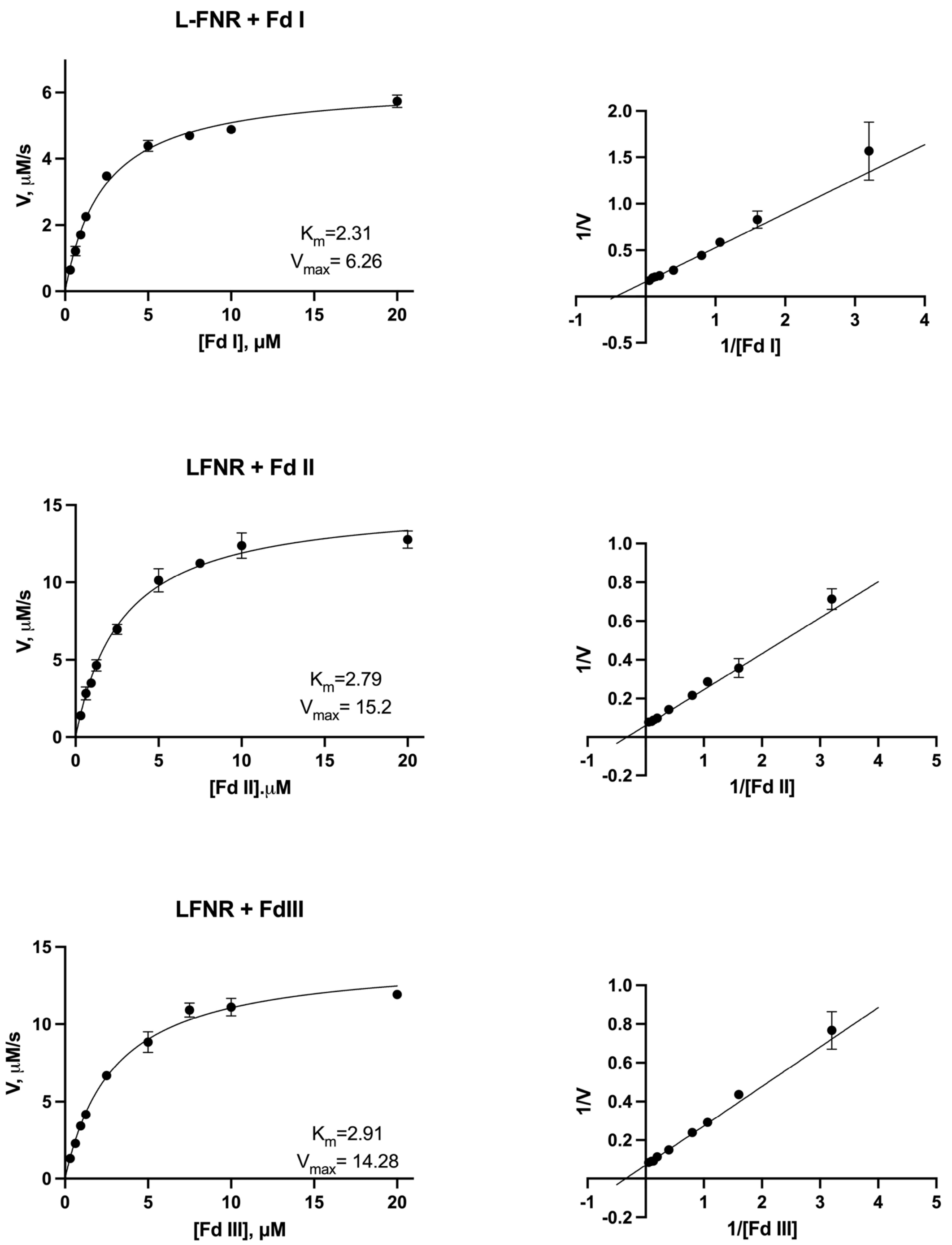

Figure S10. Cytochrome c reduction Michaelis-Menten curves (left side) and Lineweaver-Burk plots (right side) for 0.05 μM L-FNR paired with variable concentrations of Fd I, Fd II, and Fd III from peppermint.

```
                         10         20         30         40         50         60
                    ....|....| ....|....| ....|....| ....|....| ....|....| ....|....|
Maize_FdIII         MSTS----TF ATSCTLLGNV RTTQASQTAV KSPSSLSFFS QVTK---VPS LKTSKKLDVS  53
Peppermint_FdII     MAAA----SL PTICMFKSAP QRQ-TTGAFV KIPSSLGSVK STSR---IFG LKAKPDFKAT  52
Peppermint_FdIII    MATA----RL PSNCVITTAP LNKKTASAFT RGSISLGSVK SITK---TFG LKAKLDFRAS  53
Maize_FdI           MATVLGSPRA PA-FFFSSSS L--------- RAAPAPTAVA LPAAKVGIMG RSASSRRRLR  50
Peppermint_FdI      MATLSSTM-F NR-AFLTRKP L--------- AGATSLRSVN Q-AALFGL-- KSSSGGGRVT  46
Clustal Consensus   *::             :                 :   .     :          .:.        7

                             70         80         90        100        110        120
                    ....|....| ....|....| ....|....| ....|....| ....|....| ....|....|
Maize_FdIII         AMAVYKVKLV GPEGEEHEFD APDDAYILDA AETAGVELPY SCRAGACSTC AGKIESGSVD  113
Peppermint_FdII     AMAVYKVKLI GPDGDETEFE APDDCYILDS AESAGVELPY SCRAGACSTC AGKMEKGTVD  112
Peppermint_FdIII    AMATYKVKLI GADGEECEFE APDDCYILDS AETAGVELPY SCRAGACSTC AGKMVSGSVD  113
Maize_FdI           AQATYNVKLI TPEGE-VELQ VPDDVYILDQ AEEDGIDLPY SCRAGSCSSC AGKVVSGSVD  109 (60)
Peppermint_FdI      CMASYKVKLL TPEGE-VEFD CPDDMYIVDK AEEEGVDLPY SCRAGSCSSC AGKVVSGSVD  105
Clustal Consensus   . * *:***:   :*:  *::  *** **:*  **  *::*** *****:**:* ***: .*:**  53

                            130        140        150        160
                    ....|....| ....|....| ....|....| ....|....| .
Maize_FdIII         QSDGSFLDDG QQEEGYVLTC VSYPKSDCVI HTHKEGDLY- - 152
Peppermint_FdII     QSDGSFLDDK QMEEGYLLTC VSYPTADCVI HTHKEGDLY- - 151
Peppermint_FdIII    QSDGSFLDDN QMEQGYLLTC VSYPTSDCVI HTHKESDLY- - 152
Maize_FdI           QSDQSYLDDG QIADGWVLTC HAYPTSDVVI ETHKEEELTG A 150 (101)
Peppermint_FdI      QSDGSFLDDE QVAEGWVLTC VAYPTSDVVI ATHKEDDIA- - 144
Clustal Consensus   *** *:***  *  :*::***  :**.:* **  **** ::     82
```

Figure S11. Alignment of Fd sequences. Residue numbers in parentheses are counted after removal of the plastidial targeting sequence. Legend for consensus: "*" means that the residues in that column are identical in all sequences in the alignment; ":" means that conserved substitutions have been observed; "." means that semi-conserved substitutions are observed. Conserved means the amino acid is replaced by one having similar characteristics.
